# Valentino’s syndrome with secondary appendiceal perforation and fungal involvement

**DOI:** 10.1093/jscr/rjag945

**Published:** 2026-10-27

**Authors:** Nicholas Wijaya, Hanif Ardiansyah Sulistya, Wahju Sigit Tjahjono Putro, Jauhar Tajudin, Aditya Rachmat Febrianto, Clarishella Melvina Deinera, Budi Prakoso, Wisniardhy Suarnata Pradana, Muhammad Rodli, Neviana Fitri Lestari, Nayla Rahmadiani, Mohammad Agung Marzah

**Affiliations:** Emergency Department, Dr. Soepraoen Army Hospital, Jl. S. Supriadi No. 22, Sukun, Malang 65147, Indonesia; Emergency Department, Dr. Soepraoen Army Hospital, Jl. S. Supriadi No. 22, Sukun, Malang 65147, Indonesia; Department of Surgery, Faculty of Medicine, Brawijaya University, Dr. Saiful Anwar General Academic Hospital, Jl. Jaksa Agung Suprapto No. 2, Klojen, Malang 65111, Indonesia; Emergency Department, Dr. Soepraoen Army Hospital, Jl. S. Supriadi No. 22, Sukun, Malang 65147, Indonesia; Department of Surgery, Faculty of Medicine, Brawijaya University, Dr. Saiful Anwar General Academic Hospital, Jl. Jaksa Agung Suprapto No. 2, Klojen, Malang 65111, Indonesia; Department of General Surgery, Dr. Soepraoen Army Hospital, Jl. S. Supriadi No. 22, Sukun, Malang 65147, Indonesia; Emergency Department, Dr. Soepraoen Army Hospital, Jl. S. Supriadi No. 22, Sukun, Malang 65147, Indonesia; Department of Internal Medicine, Dr. Soepraoen Army Hospital, Jl. S. Supriadi No. 22, Sukun, Malang 65147, Indonesia; Department of Radiology, Dr. Soepraoen Army Hospital, Jl. S. Supriadi No. 22, Sukun, Malang 65147, Indonesia; Department of Anesthesiology and Intensive Therapy, Dr. Soepraoen Army Hospital, Jl. S. Supriadi No. 22, Sukun, Malang 65147, Indonesia; Department of Anatomical Pathology, Dr. Soepraoen Army Hospital, Jl. S. Supriadi No. 22, Sukun, Malang 65147, Indonesia; Department of Anatomical Pathology, Faculty of Medicine, Brawijaya University, Dr. Saiful Anwar General Hospital, Jl. Jaksa Agung Suprapto No. 2, Klojen, Malang 65111, Indonesia; Department of Clinical Microbiology, Faculty of Medicine, Brawijaya University, Dr. Saiful Anwar General Hospital, Jl. Jaksa Agung Suprapto No. 2, Klojen, Malang 65111, Indonesia

**Keywords:** Valentino’s syndrome, pre-pyloric perforation, secondary appendiceal perforation, aspergillus, peptic ulcer perforation, acute abdomen

## Abstract

Valentino’s syndrome (VS) is a rare presentation of perforated peptic ulcer disease causing right lower quadrant (RLQ) pain mimicking acute appendicitis. Secondary appendiceal perforation with fungal colonization is exceptionally uncommon. A 65-year-old woman presented with worsening RLQ pain, nausea, vomiting, diffuse peritoneal irritation, McBurney tenderness, and hemodynamic instability. Contrast-enhanced computed tomography demonstrated pneumoperitoneum, a pre-pyloric gastric wall defect, and periappendiceal inflammation suggestive of VS. Exploratory laparotomy confirmed generalized peritonitis from pre-pyloric gastric perforation with contamination tracking along the right paracolic gutter. A retrocecal appendix with secondary tip perforation further obscured the diagnosis. Appendectomy and primary gastric repair were performed. Histopathology showed extensive necrosis and fungal hyphae morphologically consistent with Aspergillus spp. on Grocott-Gomori methenamine silver staining, interpreted as secondary fungal involvement. This case broadens the clinical spectrum of VS and highlights careful histopathological interpretation in severely necrotic tissue.

## Introduction

Valentino’s syndrome (VS) is a rare presentation of perforated gastric or duodenal ulcer causing right lower quadrant (RLQ) pain that mimics acute appendicitis [1, 2]. The condition results from leakage of gastrointestinal contents along the right paracolic gutter, causing RLQ peritoneal irritation despite an upper gastrointestinal source [3, 4]. Delayed diagnosis may result in generalized peritonitis and sepsis [1, 5].

Fungal involvement in perforated peptic ulcer disease is uncommon [6], and secondary Aspergillus colonization in VS has rarely been reported [4]. We report a case of VS in a 65-year-old woman with histopathological findings consistent with secondary Aspergillus colonization. This case report was prepared in accordance with the SCARE 2023 guidelines.

## Case presentation

A 65-year-old woman presented with one week of RLQ pain, worsening over the preceding 3 days (Visual Analog Scale 9/10), accompanied by nausea, postprandial vomiting, and dizziness. Fever, hematemesis, melena, hematochezia, and constipation were absent. Her history included long-standing dyspepsia treated with antacids, uncontrolled hypertension, and intermittent nonsteroidal anti-inflammatory drug (NSAID) use for musculoskeletal discomfort, without known diabetes mellitus. On admission, the patient was hypotensive (79/54 mmHg) and tachycardic (112 beats/min). Abdominal examination revealed marked RLQ tenderness with positive McBurney point tenderness, guarding, and tympanic percussion.

Laboratory findings showed severe anemia (hemoglobin 6.7 g/dl), leukocytosis (13 710/μl) with neutrophil predominance, and marked hyperglycemia (random blood glucose 355 mg/dl). Initial management included fluid resuscitation, packed red blood cell transfusion, intravenous proton pump inhibitor therapy, broad-spectrum antibiotics, and glycemic control. Because the severity of diffuse peritoneal irritation and hemodynamic instability appeared disproportionate to uncomplicated appendicitis, perforated viscus was suspected.

Contrast-enhanced computed tomography (CECT) demonstrated pneumoperitoneum (Fig. 1), a focal antro-pyloric wall defect with extraluminal air consistent with perforated peptic ulcer disease (Fig. 2), as well as a thickened appendix measuring ~9.1 mm with periappendiceal inflammatory changes (Fig. 3), raising suspicion for VS.

**Figure 1:**
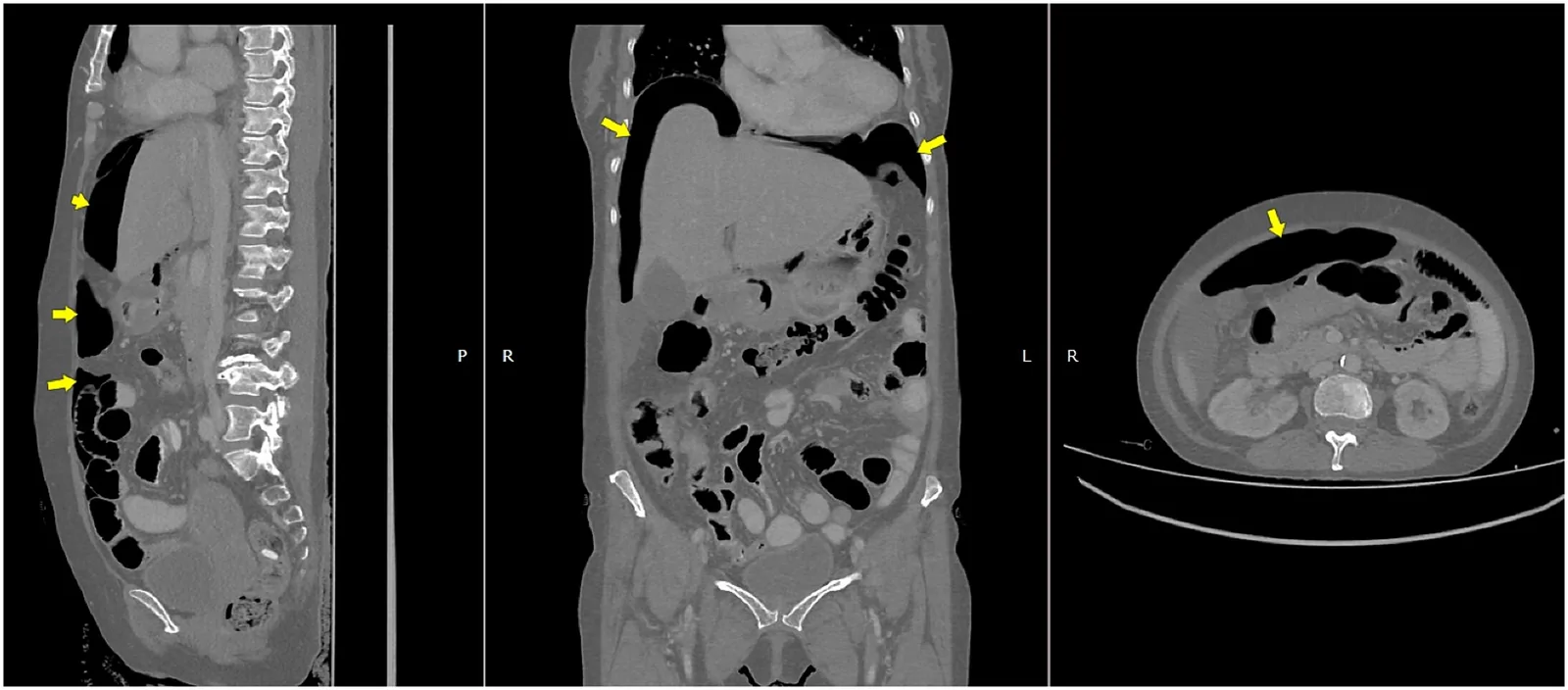
Pre-contrast CT of the abdomen, shown in sagittal (left), coronal (middle), and axial (right) views. Arrows indicates the presence of pneumoperitoneum.

**Figure 2:**
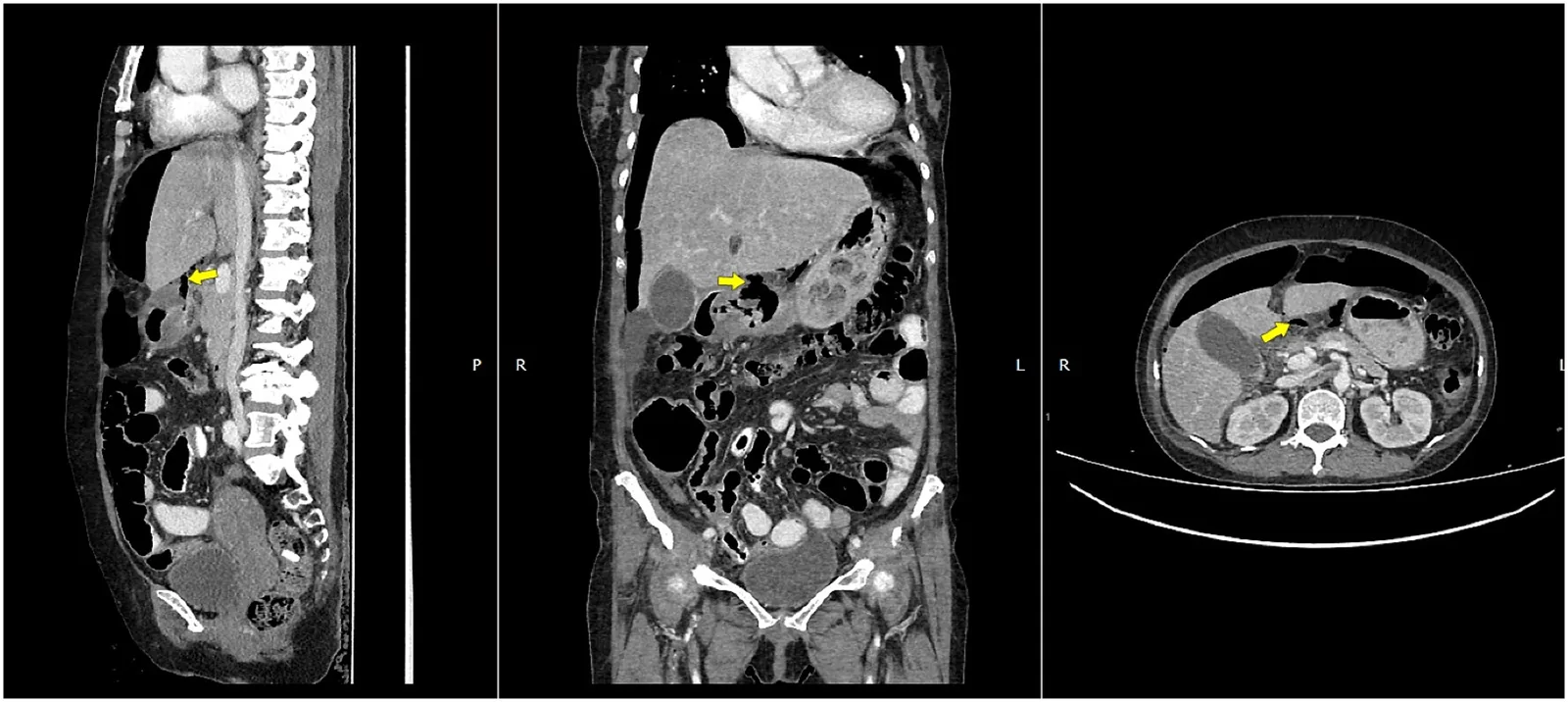
CECT of the abdomen. The arrow indicates a pyloric wall defect with extraluminal air extending into the extragastric region.

**Figure 3:**
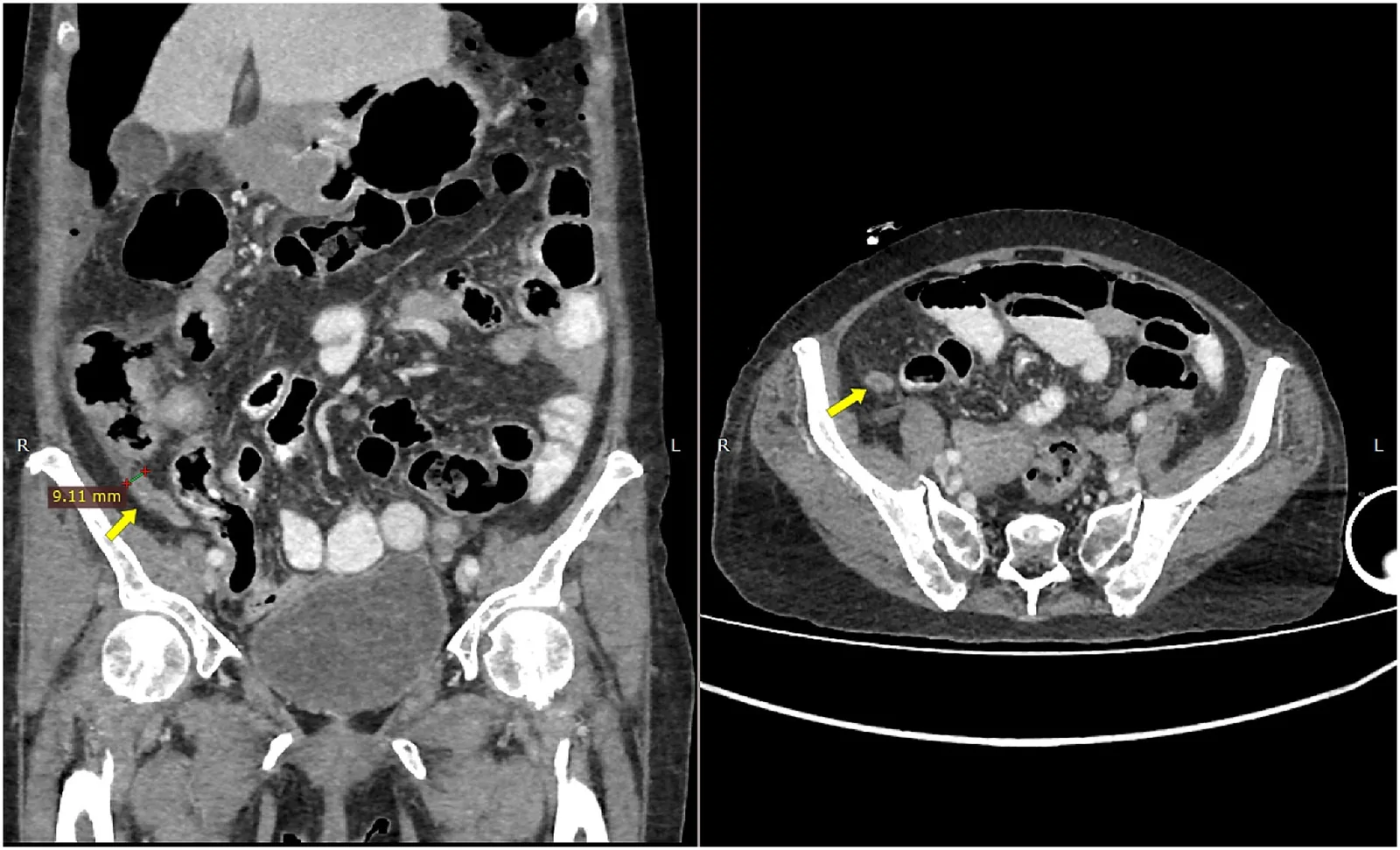
CECT of the abdomen in the venous phase, shown in coronal (left) and axial (right) views. The arrow indicates a thickened appendix measuring ~9.1 mm in diameter.

Exploratory laparotomy via a midline incision revealed turbid fluid and fibrinous exudates consistent with generalized peritonitis. A pre-pyloric gastric perforation extending to the RLQ through the right paracolic gutter was identified (Fig. 4A and B), confirming VS. No active bleeding was observed. A retrocecal inflamed appendix with tip perforation and necrotic debris indicated secondary involvement (Fig. 4C). Appendectomy with primary repair of the gastric perforation was performed, followed by lavage and drain placement in the right lesser sac (via the foramen of Winslow) and left pelvic cavity.

**Figure 4:**
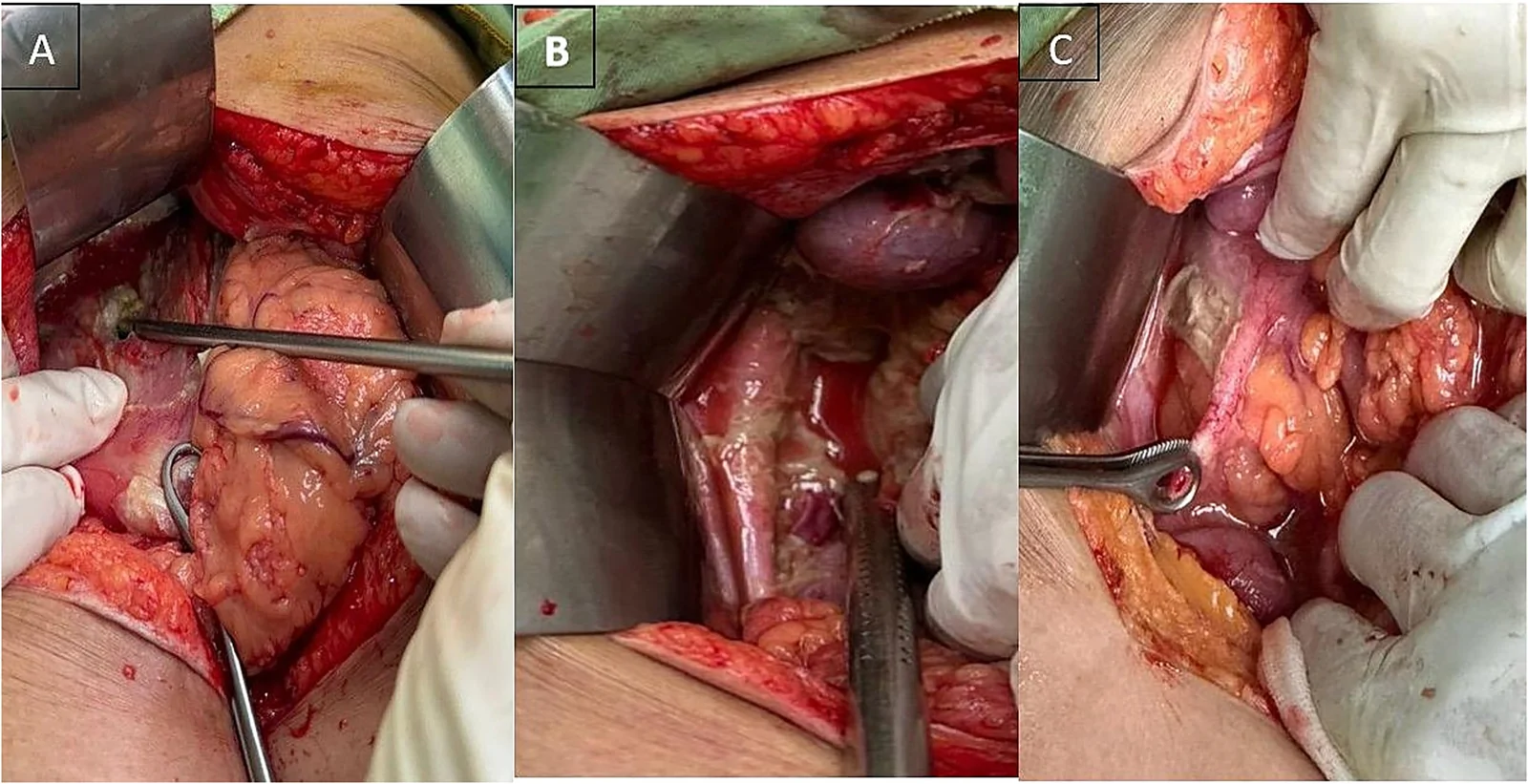
Intraoperative findings. (A) Pre-pyloric gastric perforation with adjacent inflammatory changes and fibrinous exudates. (B) Contamination tracking along the right paracolic gutter. (C) Retrocecal appendix with tip perforation and surrounding inflammatory contamination.

A biopsy specimen from the perforation margin was obtained to exclude underlying malignancy. Histopathological examination using hematoxylin and eosin staining demonstrated extensive necrosis and inflammatory infiltrates among fibrous and adipose tissue without evidence of malignancy. Fungal hyphae suspicious for *Aspergillus spp*. were identified on Gomori methenamine silver staining (GMS) (Fig. 5). As no definite tissue invasion was observed, the findings were interpreted as secondary fungal colonization rather than invasive gastric aspergillosis. Fungal culture and *Helicobacter pylori* testing were not performed because of resource limitations and the emergent operative setting. Antifungal therapy was subsequently administered following the histopathological findings.

**Figure 5:**
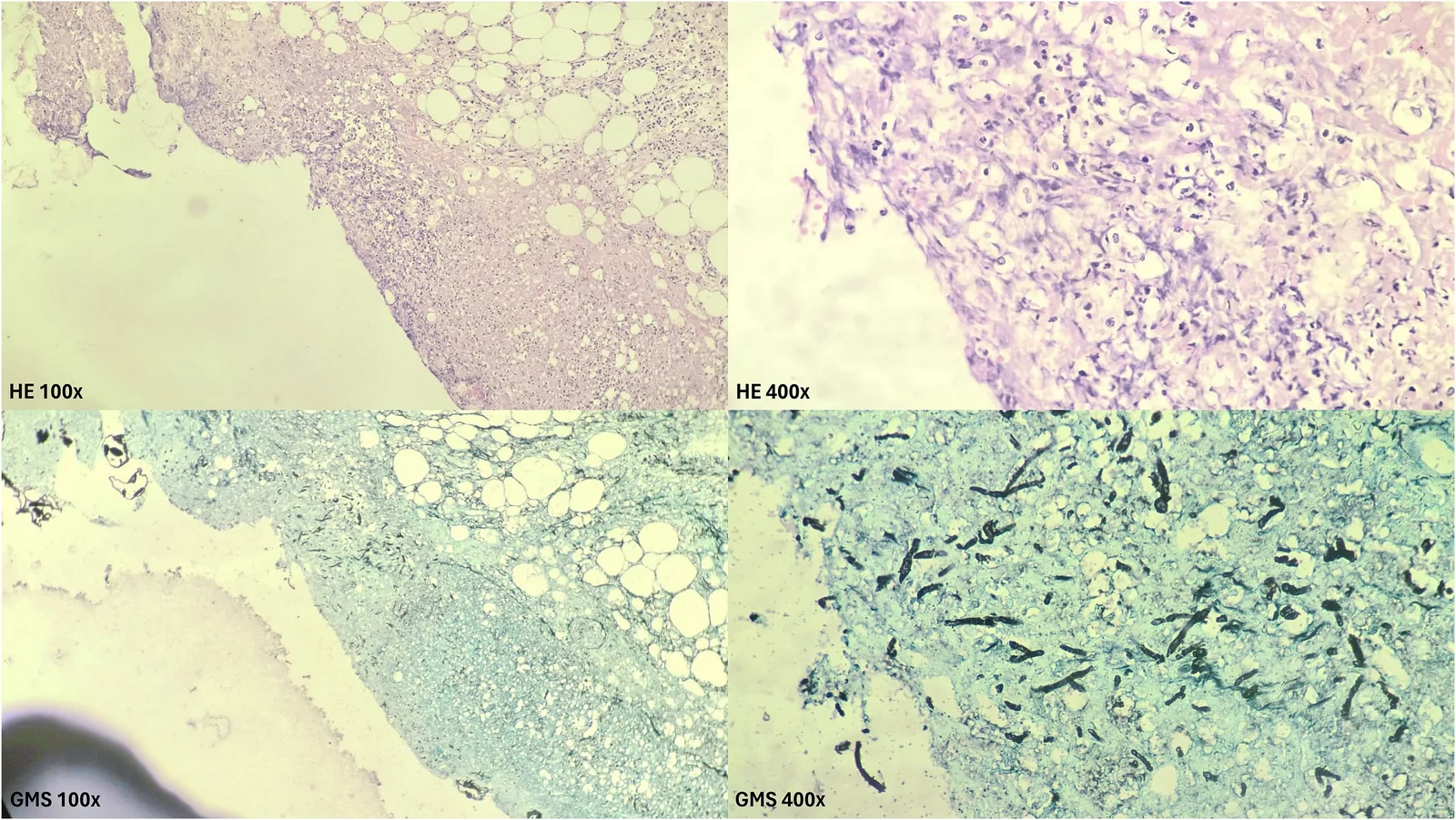
Histopathological examination of the gastric perforation specimen. Hematoxylin and eosin staining (top-left and top-right) demonstrated extensive necrosis and inflammatory infiltrates with suspicious fungal structures. GMS staining (bottom-left and bottom-right) highlighted fungal hyphae morphologically consistent with *Aspergillus spp*. within necrotic tissue. No evidence of malignancy was identified.

The postoperative course was uneventful. Drains were removed on postoperative Day 3, and the patient was discharged on Day 4 (Fig. 6). At 1-week follow-up, she remained clinically stable without complications.

**Figure 6:**
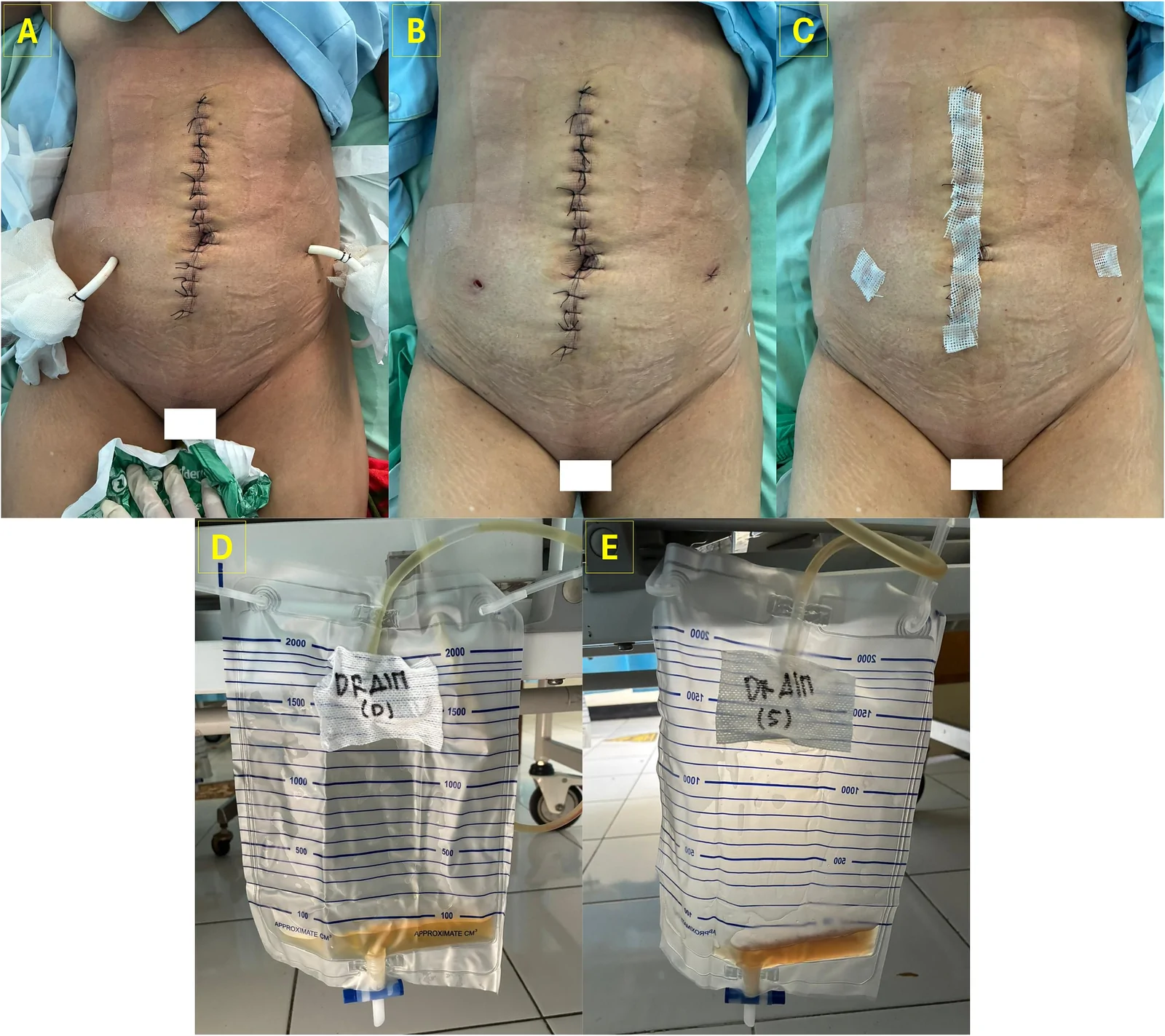
Postoperative findings. (A–C) Abdominal wound on postoperative Day 3 demonstrating satisfactory healing after removal of bilateral abdominal drains and ongoing wound care. (D and E) Collected drain output from the right lesser sac and left pelvic cavity.

## Discussion

VS is an uncommon manifestation of perforated peptic ulcer disease in which leaked gastric or duodenal contents track along the right paracolic gutter, resulting in RLQ peritoneal irritation that mimics acute appendicitis [1, 2]. Because patients commonly present with RLQ pain, McBurney tenderness, and leukocytosis, the diagnosis may initially be mistaken for primary appendicitis [3]. In our patient, diffuse guarding, tympanic percussion, hemodynamic instability, and CT findings instead suggested upper gastrointestinal perforation.

Pre-pyloric perforation was identified as the primary source of contamination in this case. Although duodenal perforation is classically associated with VS, our case demonstrated that pre-pyloric perforation is also a recognized anatomical source because leaked gastric contents may descend toward the RLQ through the right paracolic gutter [1, 4, 5]. CECT was particularly useful because it identified pneumoperitoneum, upper gastrointestinal wall defects, and inflammatory tracking toward the RLQ, all of which supported the diagnosis preoperatively [4, 7].

A particularly unusual feature of this case was appendiceal perforation. Most reported VS cases describe either a normal appendix or reactive periappendiceal inflammation without frank perforation [4, 8]. In our patient, extensive inflammatory contamination surrounded the retrocecal appendix, without appendiceal obstruction. Although primary perforated appendicitis could not be completely excluded, the presence of a definite pre-pyloric perforation with extensive contamination extending toward the RLQ favored secondary appendiceal involvement through contiguous peritoneal inflammation. Recognition of this phenomenon is clinically relevant because appendiceal perforation in the setting of VS may create the impression of primary appendicitis, potentially obscuring the actual source of contamination and complicating operative decision-making.

Another notable finding was fungal hyphae morphologically consistent with *Aspergillus spp*. identified within necrotic perforation tissue. To our knowledge, fungal involvement associated with VS has rarely been described [4]. The fungal structures demonstrated septate hyphae with acute-angle branching morphology, features commonly associated with *Aspergillus spp* [9, 10]. Several factors in our patient, including advanced age, severe anemia, marked hyperglycemia, prolonged NSAID exposure, poor nutritional status, and extensive tissue necrosis, may have contributed to impaired local host defense and secondary fungal colonization.

However, because the specimen predominantly consisted of necrotic tissue without preserved viable gastric wall architecture, angioinvasion and definitive tissue invasion could not be reliably assessed. Therefore, the findings were interpreted as secondary fungal involvement rather than proven invasive gastric aspergillosis [9]. Adjunctive antifungal therapy was administered because early invasive fungal involvement could not be excluded.

Importantly, the presence of secondary appendiceal perforation in VS may create a misleading intraoperative impression of primary appendicitis, potentially obscuring the actual source of contamination. Favorable postoperative recovery following prompt surgical source control and supportive management further emphasizes the importance of early recognition and timely intervention in atypical VS presentations.

## Conclusion

This case broadens the clinical spectrum of VS by demonstrating that upper gastrointestinal perforation may not only mimic acute appendicitis clinically but may also result in secondary appendiceal perforation. Fungal hyphae within necrotic perforation tissue highlight the importance of cautious histopathological interpretation in severely compromised tissue.
